# A randomised, multi‐centre phase III study of 3 different doses of intravenous immunoglobulin 10% in patients with chronic inflammatory demyelinating polyradiculoneuropathy (ProCID trial): Study design and protocol

**DOI:** 10.1111/jns.12267

**Published:** 2018-04-26

**Authors:** David R. Cornblath, Hans‐Peter Hartung, Hans D. Katzberg, Ingemar S. J. Merkies, Pieter A. van Doorn

**Affiliations:** ^1^ Department of Neurology Johns Hopkins University School of Medicine Baltimore Maryland; ^2^ Department of Neurology, Medical Faculty Heinrich Heine University Düsseldorf Germany; ^3^ Department of Neurology, University of Toronto Toronto General Hospital/UHN Toronto Ontario Canada; ^4^ Department of Neurology Maastricht University Medical Center Maastricht The Netherlands; ^5^ Department of Neurology Erasmus University Medical Center Rotterdam The Netherlands

**Keywords:** chronic inflammatory demyelinating polyradiculoneuropathy, intravenous immunoglobulin, randomised‐controlled trial, study design, trial protocol

## Abstract

Patients with chronic inflammatory demyelinating polyradiculoneuropathy (CIDP) show varying degrees of response to intravenous immunoglobulin (IVIg) therapy. This randomised phase III study in patients with CIDP (ProCID trial) will compare the efficacy and safety of 3 different doses (0.5, 1.0, and 2.0 g/kg) of IVIg 10% (panzyga) administered every 3 weeks for 24 weeks. The primary efficacy endpoint is the rate of treatment response, defined as a decrease in adjusted inflammatory neuropathy cause and treatment disability score of ≥1 point, in the IVIg 1.0 g/kg arm at week 24. Patients with definite or probable CIDP according to European Federation of Neurological Sciences/Peripheral Nerve Society criteria with IVIg or corticosteroid dependency and active disease are eligible. All potentially eligible patients will undergo IVIg or corticosteroid dose reduction (washout phase) over ≤12 weeks or until deterioration of CIDP (active disease). Patients with deterioration during the washout phase will be randomised to receive study treatment during a dose‐evaluation phase starting with a loading dose of IVIg 2.0 g/kg followed by maintenance treatment with IVIg 0.5, 1.0, or 2.0 g/kg every 3 weeks. Rescue medication (2 doses of IVIg 2.0 g/kg given 3 weeks apart) will be administered to patients in the IVIg 0.5 and 1.0 g/kg groups who deteriorate after week 3 and before week 18 or who do not improve at week 6. Safety, tolerability and quality of life will be assessed. The ProCID study will provide new information on the best maintenance dose of IVIg for patients with CIDP.

## 1 INTRODUCTION

Chronic inflammatory demyelinating polyradiculoneuropathy (CIDP) is an acquired peripheral neuropathy, for which first‐line therapy options include intravenous immunoglobulin (IVIg), corticosteroids, and plasma exchange.^1^ The degree of treatment response to immunoglobulin differs between patients with CIDP, and the time to and duration of maximum effect can vary from weeks to months.^2^ In the IVIg in CIDP Efficacy (ICE) study of 117 patients with CIDP, maintenance treatment with IVIg 1 g/kg every 3 weeks over 24 weeks was shown to be effective^3^; however, the European Federation of Neurological Sciences (EFNS)/Peripheral Nerve Society (PNS) 2010 guidelines recommend that the appropriate IVIg maintenance dose and frequency needs to be individualised based on the patient's treatment response, typically at 0.4 to 1.2 g/kg every 2 to 6 weeks.^1^

In almost all of the previous clinical studies of IVIg in patients with CIDP, only one dose of IVIg was assessed.^3^, ^4^, ^5^, ^6^, ^7^, ^8^, ^9^, ^10^, ^11^ The efficacy and safety of different induction doses of IVIg (0.05, 0.2, or 0.4 g/kg/d for 5 days) has been evaluated head‐to‐head in only one non‐placebo‐controlled study of Japanese patients with CIDP (*n* = 40) or multi‐focal motor neuropathy (*n* = 20), in which dose‐dependent improvements in electrophysiology and clinical disease at 5 weeks were observed in all patients.^12^ The Polyneuropathy and Treatment with Hizentra (PATH) study, which investigated 2 different maintenance doses of weekly subcutaneous immunoglobulin (SCIg) 0.2 or 0.4 g/kg in 172 patients with CIDP, showed that both doses of SCIg had significantly superior efficacy to placebo.^13^, ^14^

The ProCID study is a randomised, multi‐centre, phase III trial of IVIg in patients with CIDP that will compare the efficacy and safety of 3 different maintenance doses (0.5, 1.0, and 2.0 g/kg) of IVIg (panzyga) administered every 3 weeks over 24 weeks, following an induction dose of 2 g/kg. Data from the ProCID study will provide important new information on the best maintenance dose of IVIg to use when treating patients with CIDP. Here, we report the design and protocol of the ProCID study.

## 2 MATERIALS AND METHODS

### 2.1 Study design

**Figure 1:**
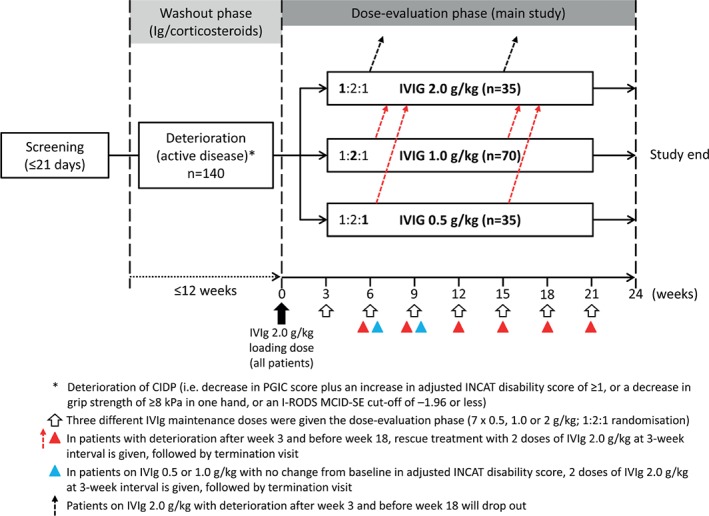
Overview of ProCID study design. CIDP, chronic inflammatory demyelinating polyradiculoneuropathy; INCAT, inflammatory neuropathy cause and treatment; I‐RODS, inflammatory Rasch‐built overall disability scale; IVIg, intravenous immunoglobulin; MCID‐SE, minimum clinically important difference‐SE; PGIC, patient global impression of change

The ProCID study is a prospective, randomised, double‐blind, parallel‐group, multi‐centre, phase III trial. An overview of the study design is shown in Figure 1. The trial started in May 2017 and is expected to be completed by the end of 2019, corresponding to a total study duration of 30 months. The study will be conducted according to the ethical principles of the Declaration of Helsinki, and in compliance with the study protocol, International Conference on Harmonisation‐Good Clinical Practice E6 regulations, and applicable regulatory requirements. Trial registration: EudraCT 2015‐005443‐14; NCT02638207.

### 2.2 Patient population

The study will enrol a minimum of 140 adult patients with definite or probable CIDP according to EFNS/PNS criteria^1^ from approximately 45 study sites worldwide. The enrolment period is expected to last for about 20 months. Eligible patients will be those who have active disease (defined below) and are dependent on IVIg or corticosteroids.

Full study inclusion and exclusion criteria are shown in Table 1.

**Table 1:** Study inclusion and exclusion criteria

Inclusion criteria
Age ≥18 years A diagnosis of definite or probable CIDP according to the EFNS/PNS 2010 guideline, including patients with MADSAM or pure motor CIDP Currently dependent on immunoglobulin or corticosteroid treatment Active disease (ie, not in remission) with progression or relapse prior to study entry or during the washout phase Weakness of at least 2 limbs Adjusted INCAT disability score of 2 to 9, with a score of 2 exclusively from leg disability Written, fully informed, voluntary consent before conduction of any study‐related procedures
Exclusion criteria
Unifocal forms of CIDP, pure sensory CIDP or MMN with conduction block Previous failure of immunoglobulin treatment Treatment with immunomodulatory or immunosuppressive agents (eg, ciclosporin, methotrexate, mitoxantrone, mycophenolate mofetil or azathioprine) within 6 months of study baseline Treatment with rituximab, alemtuzumab, cyclophosphamide, or other intensive chemotherapy, previous lymphoid irradiation or stem cell transplantation within 12 months of study baseline Respiratory impairment requiring mechanical ventilation Myelopathy or evidence of CNS demyelination or significant persisting neurological deficits from stroke or CNS trauma Clinical evidence of peripheral neuropathy from another cause such as connective tissue disease or systemic lupus erythematosus, HIV infection, hepatitis or Lyme disease, cancer (with the exception of basal cell skin cancer), or IgM paraproteinaemia with anti‐myelin‐associated glycoprotein antibodies Diabetic neuropathy, with the exception stable HbA1c (not exceeding the required normal values) in treated patients with diabetes Body mass index ≥40 kg/m^2^ Cardiac insufficiency (NHYA III/IV), cardiomyopathy, significant cardiac dysrhythmia requiring treatment, or unstable or advanced ischaemic heart disease Severe liver disease (ALT 3× greater than normal) or severe kidney disease (creatinine levels 1.5× greater than normal) Hepatitis B, hepatitis C, or HIV infection Thromboembolic events, including a history of DVT within 12 months of study baseline, any history PE, or susceptibility to DVT or PE Uncompensated hypothyroidism (abnormally high TSH and abnormally low thyroxine) or known vitamin B_12_ deficiency without adequate substitution therapy Medical conditions that may alter protein catabolism and/or IgG utilisation (eg, protein‐losing enteropathy, nephrotic syndrome) Known IgA deficiency with antibodies to IgA History of hypersensitivity, anaphylaxis or severe systemic response to immunoglobulin, blood or plasma‐derived products, or any component of panzyga Known blood hyperviscosity or other hypercoagulable states Use of other blood or plasma‐derived products within 3 months of study baseline Past or present history of drug or alcohol abuse within 5 years of study baseline An inability or unwillingness to understand or comply with the study protocol Participation in another interventional clinical study with an investigational medicinal product treatment currently or within 3 months of baseline Women who are breast feeding, pregnant, planning on becoming pregnant, or unwilling to use effective birth control during the study

ALT, alanine aminotransferase; CIDP, chronic inflammatory demyelinating polyradiculoneuropathy; CNS, central nervous system; DVT, deep vein thrombosis; EFNS, European Federation of Neurological Societies; HbA1c, glycated haemoglobin; HIV, human immunodeficiency virus; IgA/G/M, immunoglobulin A/G/M; INCAT, Inflammatory Neuropathy Cause and Treatment; MADSAM, multi‐focal acquire demyelinating sensory and motor neuropathy; MMN, multi‐focal motor neuropathy; NHYA, New York Heart Association; PE, pulmonary embolism; PNS, Peripheral Nerve Society; TSH, thyroid‐stimulating hormone.

After screening, eligible patients will undergo a predefined reduction of immunoglobulin or corticosteroids over a maximum of 12 weeks or until deterioration (washout phase). Immunoglobulin doses will be reduced by 25% at each sequential infusion, and corticosteroids will be reduced as per investigator discretion.

Patients with deterioration of CIDP during the washout phase (ie, those with active disease) will be randomised to receive study treatment. Deterioration is defined as a decrease in the patients' global impression of change (PGIC) scale plus one of the following: (1) an increase of ≥1 point on the adjusted inflammatory neuropathy cause and treatment (INCAT) disability score,^6^ (2) a decrease of ≥8 kPa in grip strength in one hand,^15^ or (3) an Inflammatory Rasch‐built Overall Disability Scale (I‐RODS) minimum clinically important difference SE (MCID‐SE) cut‐off of −1.96 or less.^16^, ^17^

Patients who do not experience deterioration of CIDP during the 12‐week washout phase (ie, those with inactive disease during the 12 weeks) will not continue in the study. During the study, the investigator may decide to withdraw patients in cases of adverse events (AEs), poor compliance, withdrawal of patient consent, pregnancy, risk of severe harm due to continued study treatment or protocol procedures, disease development that interferes with study treatment or meets an exclusion criterion, or administration of an immunoglobulin product other than panzyga. Additional patients will be enrolled when the proportion of patients who withdraw exceeds 10% (unless the planned number of patients in the dose group has already been achieved). Replacement patients will be assigned to the same dose group as the withdrawn patients; however, patients who withdraw from the study because of safety issues will not be replaced.

### 2.3 Study treatment

All patients who worsen in the washout phase will receive an initial loading dose of IVIg 2.0 g/kg. Patients will then be centrally randomised to maintenance treatment using an interactive web response system and stratified by previous immunoglobulin or corticosteroid treatment using a stratified block design. Patients will be assigned in a 1:2:1 ratio to receive double‐blind maintenance IVIg (panzyga) doses of 0.5, 1.0, or 2.0 g/kg, respectively, administered every 3 weeks (±4 days) for 24 weeks (dose‐evaluation phase).

To maintain blinding during the dose‐evaluation phase, all patients will receive the same infusion volume as in the 2.0 g/kg treatment arm, using saline solution to match the volume in the 0.5 and 1.0 g/kg treatment arms. Each infusion will be administered over 2 consecutive days, and the content of each infusion bag will be concealed with an overpouch (normally used for light protection) and new identical labels on both IVIg and saline bags. The IVIg and saline solutions will not be mixed prior to administration (ie, no dilution of IVIg will occur).

Rescue medication with 2 doses of IVIg 2.0 g/kg given 3 weeks apart may be administered to the patients in the IVIg 0.5 and 1.0 g/kg groups if they have: (1) deterioration of CIDP (defined as an increase in adjusted INCAT disability score of ≥1 point) after week 3 and before week 18; or (2) no improvement in CIDP (defined as an unchanged adjusted INCAT disability score) at week 6. After administration of rescue medication, these patients discontinue study treatment and attend an end‐of‐study assessment visit at 3 weeks after the second rescue dose. Patients in the IVIg 2.0 g/kg group with deterioration of CIDP after week 3 and before week 18 or no improvement at week 6 will also discontinue study treatment.

A summary of permitted and prohibited concomitant medications during the study is shown in Table 2.

**Table 2:** Summary of permitted and prohibited concomitant medications

Permitted medications	Prohibited medications
Stable doses of azathioprine (if receiving for ≥12 months prior to baseline) Azathioprine should be continued at the same dosage during study treatment Stable doses of corticosteroids (prednisolone or equivalent) ≤20 mg/d or equivalent in patients with prior corticosteroid therapy Paracetamol as needed for mild pain	Any other blood or plasma‐derived products (except for emergency reasons) Patients who receive immunoglobulin preparations other than panzyga will be withdrawn from the study Corticosteroids (prednisolone or equivalent) > 20 mg/d or equivalent Plasma exchange Ciclosporin, methotrexate, mitoxantrone, mycophenolate mofetil, interferon or other immunosuppressive or immunomodulatory drugs Rituximab, alemtuzumab, cyclophosphamide, or other chemotherapeutic regimens Any experimental treatment Routine pre‐medication to alleviate potential tolerability issues^a^

a Patients who experience 2 consecutive infusion‐related adverse events may receive pre‐medication with antipyretics, antihistamines, or antiemetic agents.

### 2.4 Study endpoints

The primary efficacy endpoint is the proportion of patients in the IVIg 1.0 g/kg group with response to treatment at week 24 (defined as a decrease in adjusted INCAT disability score of ≥1 point). The adjusted INCAT disability score differs from the standard score by exclusion of changes in upper limb function from 0 (normal) to 1 (minor symptoms) or from 1 to 0, as these changes are not considered to be clinically relevant in all patients.^3^

The following secondary efficacy endpoints will also be assessed: proportion of patients in the IVIg 0.5 and 2.0 g/kg groups vs the IVIg 1.0 g/kg group with response to treatment at week 24 based on the adjusted INCAT disability score, grip strength (assessed by Martin Handheld Vigorimeter, Gebrüder Martin GmbH & Co. KG, Tuttlingen, Germany) using the MCID cut‐off of 8 kPa, and I‐RODS score using the MCID‐SE cut‐off of −1.96 or less^16^; the time to first confirmed worsening (ie, increase from baseline by ≥1 point) in the adjusted INCAT disability score; the time to improvement (ie, 1 point decrease from baseline) in adjusted INCAT disability score; the time to first confirmed worsening from baseline in I‐RODS score; the time to decrease in I‐RODS score; mean change from baseline to end‐of‐study assessment visit in grip strength of both hands, I‐RODS score, Pain Intensity Numeric Rating Scale (PI‐NRS), and sum of the distal evoked amplitude of 4 right‐sided and 4 left‐sided motor nerves (peroneal, tibial, ulnar, and median); and the absolute number of improvers in grip strength and I‐RODS during the study.

Exploratory efficacy endpoints will include the mean change from baseline to study end in Modified Fatigue Severity Scale (FSS), number of patients with improvement of ≥4 points in Medical Research Council (MRC) sum score at week 12 or at time of rescue medication, and Short‐Form 36‐item (SF‐36) health survey physical component score, mental component score, and their 8 health domains; and the time to decrease in MRC sum score to baseline (or below) after temporary increase.

Safety endpoints will be assessed throughout the 24‐week dose‐evaluation phase and will include the incidence of all AEs; short‐term tolerance parameters, including vital signs; physical and neurological examination findings; and laboratory parameters (haematology and clinical chemistry) and tests for viral safety.

**Figure 2:**
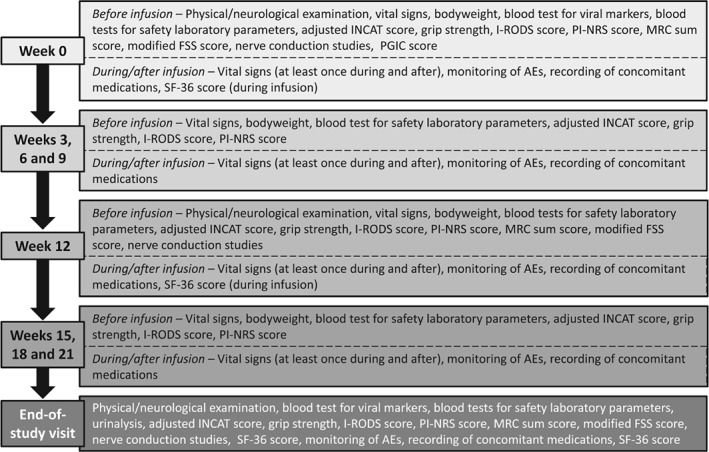
Schedule of study visit assessments during the dose‐evaluation phase. AEs, adverse events; FSS, fatigue severity scale; INCAT, inflammatory neuropathy cause and treatment; I‐RODS, inflammatory Rasch‐built overall disability scale; MRC, Medical Research Council; PGIC, Patients' global impression of change; PI‐NRS, pain intensity numeric rating scale; SF‐36, short form 36 items health status

The schedule of assessments performed at each study visit is summarised in Figure 2.

### 2.5 Statistical analysis

The statistical analysis will be delegated under an agreement of transfer of responsibilities to an external contract research organisation.

Calculation of the sample size is based on the proportion of patients with treatment response in the IVIg 1.0 g/kg group. Based on previous trials,^3^, ^7^ a threshold of 42% responders was chosen for the evaluation of the primary endpoint. To achieve a power of ≥80%, a minimum of 62 evaluable patients in the IVIg 1.0 g/kg group is needed. Therefore, the study plans to enrol 70 patients in this group to account for possible dropouts. For the comparison between doses, half of the eligible patients will be enrolled in the standard dose group (1.0 g/kg) and the other half in the lower (0.5 g/kg) and higher (2.0 g/kg) dose groups. The total target enrolment is 140 patients.

Statistical evaluation of the primary and secondary endpoints will be presented for each randomisation stratum (ie, prior treatment with IVIg or corticosteroids) separately. No further subgroup analyses are pre‐specified. Further comparisons between CIDP variants may be added post hoc as indicated by the data.

The statistical analysis will consider the safety set (SAF), full analysis set (FAS), and per‐protocol set (PPS) patient populations (Table 3). The primary endpoint analysis will be conducted for the FAS and PPS, the secondary endpoints will be evaluated in the FAS and in the PPS (in case the 2 populations differ by >5%), and the safety analysis will be conducted in the SAF. All efficacy and safety analyses will be summarised using descriptive statistics.

**Table 3:** Definitions of study patient populations

Patient population	Definition
SAF	All randomised patients who receive at least part of one infusion of study treatment
FAS	According to the intent‐to‐treat principle, and includes all patients from the SAF population for whom data were collected post‐infusion of study treatment
PPS	A subset of the FAS, which excludes patients with significant protocol deviations that could potentially significantly affect evaluation of the primary outcome^a^

FAS, full analysis set; PPS, per‐protocol set; SAF, safety set.

a The classification of protocol violations will be conducted and documented before the database is locked, the data is unblinded and the statistical analyses are performed.

In general, data derivations will be based on observed values only, and missing data will not be imputed. If missing values occur in the confirmatory analysis of the FAS primary endpoint, they will be imputed as worst observed values (ie, observed patients will be analysed as a non‐responder).

## 3 DISCUSSION

The ProCID study is a randomised, double‐blind, multi‐centre, phase III trial that has been designed to compare 3 different IVIg maintenance doses for the treatment of patients with CIDP: 0.5, 1.0, and 2.0 g/kg once every 3 weeks for 24 weeks. The use of 3 maintenance dose groups in the dose‐evaluation phase will allow for confirmation of the clinical efficacy of IVIg observed in previous clinical trials.^3^, ^4^, ^5^, ^6^, ^7^, ^8^, ^9^, ^10^, ^11^, ^12^

In a post hoc analysis of the ICE study, almost half of IVIg responders showed improvement in adjusted INCAT disability scores within the first 3 weeks of treatment (ie, after the initial 2.0 g/kg loading dose).^18^ To be comparable with data from the ICE study,^3^ the primary endpoint of the ProCID study will be the proportion of patients in the IVIg 1.0 g/kg group with response to treatment, as defined by an improvement from baseline of ≥1 point on the adjusted INCAT disability score. Consistent with the ICE^3^ and Privigen Impact on Mobility and Autonomy (PRIMA)^7^ studies, the primary endpoint of this study will be assessed at week 24.

The only other randomised‐controlled study to have prospectively evaluated the efficacy of different IVIg doses observed a dose‐response relationship following a single dose of IVIg 0.25, 1.0 or 2.0 g/kg over 5 days.^12^ Among 60 patients with CIDP or multi‐focal motor neuropathy who received treatment, high‐dose IVIg therapy (2.0 g/kg) was associated with a higher initial response rate compared with the 0.25 and 1.0 g/kg doses (response rates of 60% vs 15% and 21%, respectively) at 5 weeks; 24‐week data were not presented in this study.^12^ However, this study was not randomised and did not include a placebo group.^19^ In the PATH study of 172 patients with definite or probable CIDP who received weekly SCIg 0.2 or 0.4 g/kg or placebo, the proportion of patients who had a relapse was 19% with SCIg 0.4 g/kg and 33% with SCIg 0.2 g/kg.^14^ Although the difference in relapse rates between SCIg doses was not significant, relapse rates with both SCIg doses were significantly lower than with placebo (56%; *P* < .001).^14^

The ProCID study will also evaluate whether different maintenance doses of IVIg may result in changes in treatment response in patients with definite or probable CIDP, and whether this efficacy will differ compared with that observed in the ICE^3^ and PRIMA^7^ studies. While maintenance doses of IVIg vary considerably, most patients start with a standard maintenance dose of 1 g/kg every 3 weeks (or equivalent), with subsequent dose adjustments being made as needed. This study will evaluate whether a higher or lower starting dose to the maintenance phase is better than the widely used standard dose of 1 g/kg every 3 weeks and will assess the safety and tolerability of these additional doses to provide clinicians further data in choosing IVIg maintenance doses for patients with CIDP.
